# Bioengineering of Pulmonary Epithelium With Preservation of the Vascular Niche

**DOI:** 10.3389/fbioe.2020.00269

**Published:** 2020-04-15

**Authors:** N. Valerio Dorrello, Gordana Vunjak-Novakovic

**Affiliations:** ^1^Department of Pediatrics, Columbia University, New York, NY, United States; ^2^Department of Biomedical Engineering, Columbia University, New York, NY, United States; ^3^Department of Medicine, Columbia University, New York, NY, United States

**Keywords:** lung, bioengineering, regeneration, lung vasculature, lung epithelium, lung support *ex vivo*, decellularization, recellularization

## Abstract

The shortage of transplantable donor organs directly affects patients with end-stage lung disease, for which transplantation remains the only definitive treatment. With the current acceptance rate of donor lungs of only 20%, rescuing even one half of the rejected donor lungs would increase the number of transplantable lungs threefold, to 60%. We review recent advances in lung bioengineering that have potential to repair the epithelial and vascular compartments of the lung. Our focus is on the long-term support and recovery of the lung *ex vivo*, and the replacement of defective epithelium with healthy therapeutic cells. To this end, we first review the roles of the lung epithelium and vasculature, with focus on the alveolar-capillary membrane, and then discuss the available and emerging technologies for *ex vivo* bioengineering of the lung by decellularization and recellularization. While there have been many meritorious advances in these technologies for recovering marginal quality lungs to the levels needed to meet the standards for transplantation – many challenges remain, motivating further studies of the extended *ex vivo* support and interventions in the lung. We propose that the repair of injured epithelium with preservation of quiescent vasculature will be critical for the immediate blood supply to the lung and the lung survival and function following transplantation.

## Lung Disease and the Need for Lung Bioengineering

Nearly 25 million people suffer from end-stage lung disease in the United States alone, with a staggering ∼400,000 patients dying each year. Lung transplantation, the *only* definitive treatment for these patients, remains constrained by the severe shortage of donor organs, as only one out of five donor lungs meets the historical criteria for transplant proposed in the 1980s (including: donor age between 20 and 45 years, arterial partial pressure of oxygen (PaO_2_)/fraction of inspired O_2_ (FiO_2_) >350, no smoking history, clear chest X-ray, less than five ventilation days, clear bronchoscopy, negative gram stain of tracheal secretions, ischemic time < 4 h) (Bhorade et al., 2000; Ware et al., 2002; Filosso et al., 2006; Botha et al., 2008; Kotloff and Thabut, 2011; Valapour et al., 2020). The indications for lung transplantation have broadened over time and now include a diverse spectrum of pulmonary diseases of the airway, parenchyma, and vasculature. Chronic obstructive pulmonary disease (COPD), idiopathic pulmonary fibrosis (IPF), and cystic fibrosis (CF) are still the major indications for transplantation, whereas vascular disease such as idiopathic pulmonary arterial hypertension (IPAH) has become a lower indication (Kotloff and Thabut, 2011; Valapour et al., 2020). However, the need for lung transplant continues to exceed the availability of donor organs. Each year approximately 25% of listed patients either die or are too sick to undergo transplant and are removed from the waiting list (Keeshan et al., 2015; Valapour et al., 2020). Unfortunately the graft shortage persists and it is becoming clear that empirical criteria for donor selection are too stringent. Over the last decade, several studies have suggested that there is little impact of the historical selection criteria on lung transplant outcomes. Therefore, many centers have liberalized these criteria into what are now called “extended criteria,” allowing to increase the number of suitable lung donors up to 30–40% (Meers et al., 2010; Kotecha et al., 2017). Some examples of these criteria include donor age within 18 and 64 years, PaO_2_/FiO_2_ < 300, smoking history, abnormal chest X-ray, more medical comorbidities, ischemic time < 7 h, drug abuse, donation after cardiac death (Chaney et al., 2014; Young and Dilling, 2019). Nevertheless, an overall donor shortage remains and many lung transplant candidates do not undergo transplantation. Along with the extended criteria of donor selection and *ex vivo* reconditioning of marginal lungs, new and more effective therapeutic interventions for lung disease and transplantation are urgently needed.

The lung is an extremely complex organ, featuring intricate 3-D architecture, diversity of cellular composition (with more than 40 cell types) (Colby et al., 2007; Franks et al., 2008; Beers and Morrisey, 2011; Wagner et al., 2013), a highly specialized matrix, and the specific architectures and functions of the airway and vasculature. It is not yet possible to bioengineer a functional lung from pulmonary cells and scaffolds, even with our best technologies. Lung ventilation, constituted of inspiration and expiration, brings in oxygen (O_2_) and removes carbon dioxide (CO_2_) from circulating blood, through the process called gas exchange, the main function of the lung that occurs in the alveolar spaces. In addition to the skin, the lung is the only organ in direct contact with the external environment. Before reaching the alveoli, air passes through the conductive airways, where it gets filtered by the host physical barriers and cleared by the immune system.

**FIGURE 1:**
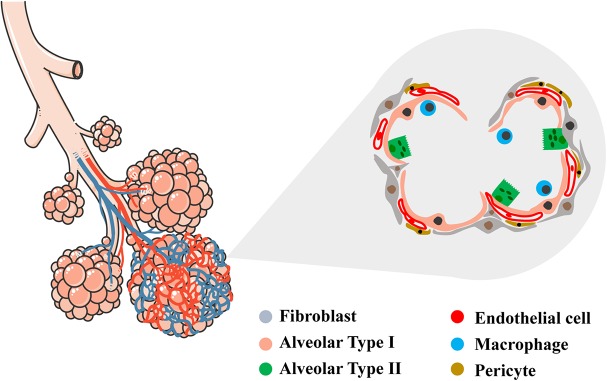
Schematic representation of conductive and respiratory zones of the lung. Tracheal, bronchial and alveolar regions are showed with their vascular network. In the magnified area, detailed representation of the alveolar region showing the diversity of epithelial, vascular and mesenchymal cells within (alveolar type I, alveolar type II, endothelial cell, fibroblast, pericyte, macrophage). Left schematic was adapted by servier medical art website (smart.servier.com).

The alveolar region of the lung (parenchyma) comprises about 90% of its volume; the other 10% consists of conducting airways and larger vessels. The air that reaches the alveoli is separated from the blood perfusing the lung by a three-layer structure: (a) the alveolar epithelium lining (alveolar type I, ATI, and alveolar type II, ATII cells), (b) connective tissue (the alveolar-capillary membrane, ACM), and (c) the endothelial lining of the alveolar capillaries, where venous blood coming from the pulmonary arteries flows to capture O_2_ and release CO_2_. In the alveoli, ATI cells – the thinnest cells in the human body – cover approximately 95% of the total alveolar surface (Figure 1). AT1 cells are interspaced by cuboidally shaped ATII cells. Even though ATII cells cover only 5% of the alveolar surface, there are twice as many ATII cells as ATI cells (Crapo et al., 1982).

In the alveolar spaces, not only do the epithelial cells come in contact with life-sustaining O_2_, but they can also be reached by airborne particles, toxins, and microbes that bypass the conductive airways – these insults can severely injure the epithelial cells. Notably, most lung diseases affect the epithelium (Table 1), prompting the need to develop bioengineering modalities for its regeneration. An equally important requirement is the development of bioengineering modalities for maintenance of the functional vasculature, which is critical for lung viability and function. To achieve these goals, highly innovative approaches are needed, due to the enormous complexity of the lung.

**TABLE 1:** List of primary epithelial and vascular lung diseases.

**Primary epithelial diseases**	**Primary vascular diseases**
Acute Respiratory Distress Syndrome (ARDS)	Pulmonary hypertension
Chronic obstructive pulmonary disease (COPD); emphysema	Pulmonary arterial hypertension Pulmonary hypertension, not idiopathic
Cystic fibrosis (CF)	Swyer-James syndrome
Interstitial lung disease (idiopathic and not)	
Other:	
Bronchopulmonary dysplasia	
Alpha-1 antitrypsin deficiency	
Surfactant proteins (B/C) deficiency	
ABCA3 mutation	
Primary ciliary dyskinesia	
Niemann-Pick disease	
Birt-Hogg-Dubé syndrome	
Lung cancer	

Lung bioengineering can bring enormous benefits to two areas of pulmonary medicine: (1) regeneration of injured or diseased lungs; and (2) bioengineering of the lung as a platform for modeling lung regeneration, disease and drug discovery. Together, these two areas aim to reduce the burden of end-stage lung disease.

In this review, we focus on recent advances in lung bioengineering aiming to repair the epithelial and vascular compartments of the lung, and in particular on bioengineering methodologies that allow preservation of the integrity and function of lung vasculature.

## The Role of Vasculature in Lung Disease and Repair

Blood vessels are extremely important in the architecture of every organ of the body, and especially in organs as highly vascularized as the lungs. The lung vascular network represents the largest microvascular network in the body, with a capillary network of endothelial cells that connects to the large arteries and veins. The capillary network is a barrier-forming cell lining and a conduit that delivers O_2_ and nutrients to the organs, and regulates coagulation cascade and metabolism between organs. In addition, the endothelial cells are responsible for vital physiological activities involved in the homeostasis and metabolism of the surrounding tissue during lung development, repair and regeneration. During development but also in adulthood, endothelial cells are regulated by organotypic transcription factors that inform the transition into organ-specific endothelial cells.

A close look at the organs’ architectures, from brain to liver to lung, shows that the endothelial cells reside very close to the parenchymal cells with dynamic cell interactions through paracrine factors. The tissue-specific cells regulate activation and response of endothelial cells and produce angiocrine factors with specific roles in organ homeostasis, regeneration, and repair. The term “angiocrine” was created to underline the instructive biological role that these factors have in the organ homeostasis (Butler et al., 2010). It is increasingly recognized that the endothelial cells have different phenotypes in different organs or even in different regions of the same organ. Endothelial cells in the brain are different from those in the liver, kidney, or lung. Recent studies have demonstrated the ability to generate *in vitro* different types of tissue-specific vasculature (Grigoryan et al., 2019). A concept of organotypic capillary endothelial cells as specialized niche cells for each organ is also emerging (Rafii et al., 2016). The angiocrine profiles of endothelial cells in different organs are quite different, making each endothelial cell subtype organ-specific. Therefore, it becomes important to identify the organ-specific angiocrine profiles modulating the function of specific stem cells.

To allow gas exchange, the pulmonary capillary endothelial cells (PCECs) of the capillary network are in proximity with the alveolar epithelial cells (Bhattacharya, 2005; Voelkel et al., 2006), with the ACM between them that is considerably different from the membranes in other vascular networks in the body (Townsley, 2012; Weibel, 2013). The close association between PCECs and epithelial cells facilitates the crosstalk between the airway and vasculature and modulates pulmonary processes.

PCECs have a distinct phenotypic signature (VEGFR2 + FGGR1 + CD34 + VE-cadherin+) that makes them distinguishable from all other cell subtypes (Ding et al., 2011). During development, lung blood vessels are actively involved in alveolar growth and the maintenance of alveolar architecture throughout postnatal life. In particular, PCECs are specialized in driving the alveolarization (DeLisser et al., 2006). Several angiogenic growth factors are essential for the proper formation of blood vessels in the lung (Bertoncello, 2015). Vascular endothelial growth factor (VEGF), the specific mitogen for vascular endothelial cells, has been found to play a pivotal role during lung regrowth post-pneumonectomy (Ding et al., 2011).

The importance of VEGF during embryogenesis has been documented in experiments with genetic inactivation of VEGF. VEGF isoforms 120, 164, and 188 are highly expressed in ATII cells during mouse lung development, especially during the canalicular stage when the angiogenesis occurs (Ng et al., 2001). Mice with targeted deletion of the VEGF gene display several vascular defects, most importantly significant reduction of air spaces and alveolar capillary, showing distended and rudimental alveoli (Galambos et al., 2002). In adult rats, treatment with VEGF receptor antagonists leads to air space enlargement, similar to that seen in emphysema (Kasahara et al., 2000).

Another set of factors shown to play a role in lung alveolarization and angiogenesis is the set of Hypoxia Inducible Factors (HIFs) (Rajatapiti et al., 2008). HIF-1, HIF-2, HIF-3 are highly expressed during lung development in epithelial cells, and the inhibition of these factors results in defective vascular branching in the lung (Compernolle et al., 2002). A clear link between angiogenesis and alveolarization has emerged from a neonatal disease, broncho-pulmonary dysplasia (BPD), where alveolar hypoplasia and dysmorphic lung vasculature co-exist and the development of lung vasculature and alveolar spaces are altered in animal models (Roberts et al., 1983; Wilson et al., 1985). Also, VEGF and HIFs levels are low in both the infants with BPD and animal models (Maniscalco et al., 1997; Bhatt et al., 2001; Thébaud et al., 2005; Vadivel et al., 2014). These observations underline the roles of VEGF and HIF in the orchestration of alveolarization and angiogenesis during lung development.

During lung development, the capillary bed sprouts together with the alveolar budding, allowing for the formation of the ACM (Cardoso, 2001; Metzger et al., 2008). This process persists during adult lung regeneration (Ding et al., 2011), where PCECs are necessary for epithelial function. After unilateral pneumonectomy in mice, Ding et al. showed that the remaining PCECs produced angiocrine growth factors to induce proliferation of epithelial progenitor cells to support alveologenesis through Vascular Endothelial Growth Factor Receptor 2 (VEGFR2) and Fibroblast Growth Factor 1 (FGFR1) (Ding et al., 2011). Conversely, endothelial-specific ablation of *Vegfr2* and *Fgfr1* after pneumonectomy impaired alveolarization and propagation of ATII cells (Ding et al., 2011). Further studies showed that, with pneumonectomy, recruitment of platelets induces release of Stromal cell-Derived Factor 1 (SDF-1) from the platelets themselves and increases production of Matrix Metallopeptidase 14 (MMP14) from PCECs with activation of ATII cells (Rafii et al., 2015). MMP14 was shown to induce basal cell proliferation (Rock et al., 2009). In a 3-D co-culture of endothelial and bronchoalveolar stem cells, Lee et al. showed that Bone Morphogenic Protein 4 (BMP4) – Bone Morphogenic Protein Receptor 1a (Bmpr1a) signaling triggers calcineurin/Nuclear Factor of Activated T cells 1 (NFATc1) – dependent expression of thrombospondin-1 (Tsp1) in PCECs to drive alveolar lineage-specific differentiation of bronchioalveolar stem cells (Lee et al., 2014). Also, Tsp1-null mice that underwent pneumonectomy exhibited impaired repair from alveolar injury and a severe reduction in both ATII cells and PCECs – confirming a crucial role for the BMP4-NFATc1-TSP1 axis in lung epithelial differentiation and alveolar regeneration (Lee et al., 2014). More importantly, the minimal contribution of the plasma from pneumonectomized mice to alveolarization shows the importance of local PCECs (Ding et al., 2011; Gomez-Salinero and Rafii, 2018).

Endothelial-epithelial crosstalk has also been demonstrated in bronchioalveolar stem cell (BASC) differentiation. BASCs are a small cell population localized at the bronchioalveolar duct junction (BADJ) in the mouse and capable of repopulating both the airway and the alveolar compartments after injury (Kim et al., 2005; Liu et al., 2019; Salwig et al., 2019). In a 3-D co-culture model of BASCs and endothelial cells, it has been shown that BMP4 can induce in endothelial cells the activation of calcineurin and secondary expression of TSP1 that ultimately drive alveolar lineage-specific BASC differentiation (Lee et al., 2014). *In vivo*, Tsp1 -/- mice exhibited defective alveolar injury repair following bleomycin injury, confirming the crucial role for the BMP4-NFATc1-TSP1 axis in lung epithelial differentiation and regeneration. These findings suggest that tight coordination between endothelial and epithelial cells is fundamental for alveolar regeneration, *in vitro* and *in vivo*.

## The Alveolar-Capillary Membrane

The alveolar-capillary barrier consists of the bipolar layer of epithelial and endothelial cells, separated by a thin membrane of specialized extracellular matrix called ACM. On the airway side, there is a continuous alveolar epithelium made of ATI and ATII cells; on the capillary side, there is a continuous PCECs lining. The continuity and tightness of the alveolar epithelium and the capillary endothelium are essential for fluid and ion transport between the alveolar and vascular spaces. The ACM is not only a fundamental structural component of the respiratory zone of the lung but also regulates – together with the adjacent cells – gas exchange, fluid homeostasis, microenvironment sensing and cell-cell interaction and migration (Kalluri, 2003; Nishiguchi et al., 2017).

The ACM consists of large insoluble molecules that form a sheet-like mesh, and are regulated by anchor proteins and receptors (Yurchenco et al., 1986; Kalluri, 2003). Looking at a macroscale picture, from the single alveolus to the respiratory zone of the lung containing millions of alveoli, the ACM is able through its unique structure and properties to build an organ as complex and important as the lung. The natural ACM of the human lung, produced by local lung cells, forms a 0.1–2 μm thick membrane (Gehr et al., 1978). The thick side of the ACM is where the cell nuclei and fiber network are concentrated, providing mechanical stability and regenerative potential, while the thin side is where the alveolar epithelium and capillary endothelium share the same basal lamina membrane. In human lung, one half of the ACM is thin, underscoring the fragility of the ACM as well as its strength to support an organ that during inflation contains 80% air, 10% tissue, and 10% blood (Knudsen and Ochs, 2018). Although epithelial, endothelial, and interstitial cells can generate forces, the contribution of the lung extracellular matrix (ECM) dominates, as shown by only minimal changes in the strain and elastic recoil of the lung ECM after decellularization (Nonaka et al., 2014). Each alveolus and its ACM have a certain elastance and viscoelasticity that collectively define mechanical properties at the organ scale.

Mass spectrometry proteomics has allowed characterization of the matrisome, or the total amount of proteins in the ECM (Hynes and Naba, 2012; Mayorca-Guiliani et al., 2017). The core proteins of the lung include type IV collagen, laminin, fibronectin, heparan sulfate proteoglycan (HSPG or perlecan), and chondroitin sulfate proteoglycan (CSPG) that form a mesh-like structure. ECM-associated proteins include nidogen/entactin, hyaluronate, tenascin X and C, thrombospondin, and agrin (Kalluri, 2003; Balestrini and Niklason, 2015; Zhou Y. et al., 2018). These components are interconnected to allow selective transfer of cells and signaling molecules, regulating tissue development, homeostasis, and disease pathogenesis (Bottaro et al., 2002; Vorotnikova et al., 2010; Otranto et al., 2012). An interesting feature of the ACM – and of all basement membranes – is the capacity to be produced by the local cells and to self-assemble into an intricate sheet-like structure that cannot be replicated using synthetic materials. Specifically, type IV collagen and laminin contain the peptide signals that allow them to initiate intermolecular self-assembly and form mesh-like structures (Cheng et al., 1997; Yurchenco et al., 2002).

Type IV collagen, a key structural factor of the ACM, provides barrier function between epithelial and endothelial cells (Dunsmore and Rannels, 1996). Fibronectin, a cell-adhesive glycoprotein, mediates the interaction of lung cells through integrins, regulating their morphology and cell behavior (Lesur et al., 1996; Kalluri, 2003). Fibronectin also serves as storage for growth factor inside “ECM pockets”, which is especially important during remodeling and regeneration (Serini et al., 1998; Balestrini and Niklason, 2015). In the alveolar region, proteoglycans (PGs) are pivotal for the retention of water, ions, and growth factors, and their localization in the ACM is critical for control of cellular movement and differentiation. For example, sulfated PGs allow differentiation and elongation of ATII versus ATI cells (Sannes, 1991; Li et al., 2000). Lung cells are differently localized on the ACM according to the ECM components. ATII cells migrate more toward fibronectin (forming in proximity to its cell-cell junctions) and less to laminin or collagens (Balestrini and Niklason, 2015). From the vascular side, endothelial cells co-localize more with fibronectin than collagen subtypes, which are preferred instead by the interstitial cells, such as fibroblasts and smooth muscle cells (Murray et al., 1980; Balestrini and Niklason, 2015).

Two characteristics of the ACM that are fundamental for bioengineering of all organs are the matrix topography and stiffness (Zhou Y. et al., 2018). Matrix topography includes the organization, size, and geometry of the matrix that translates specific signals to the cells mediating their shape, morphology, and function at the micro- and nanoscale (Bettinger et al., 2009). One example of matrix-cell interactions includes integrins, which were first identified as receptors to laminin and type IV collagen in the matrix, linking cell cytoskeleton to matrix signals (Sheppard, 2000; Kalluri, 2003). Integrins mediate signals that regulate cell behavior and gene expression through changes in adhesion, cytoskeleton and nuclear remodeling (Hynes, 2002; Dalby et al., 2003). Matrix stiffness is another important signal for the cells to generate traction and activate the TGF-ß pathway that regulates the production and stiffness of new matrix (Wipff et al., 2007). Matrix stiffness can become abnormal in diseases like emphysema or IPF (Zhou Y. et al., 2018). In IPF, alveolar epithelial injury induces basement membrane disruption and activation of fibroblasts that remodel the local matrix, forming a scar rich in fibronectin and collagen. When this process becomes exaggerated, there is fibrosis and loss of epithelial cells (Thannickal et al., 2014). Identification of signals released by ACM would allow better understanding of engraftment and differentiation of lung progenitors on decellularized lungs and gain new insights into how pathological changes in ACM (e.g., in IPF and COPD) alter stem cell behavior and regenerative capacity.

## Mechanical Stimuli in Lung Regeneration

The lung and its key unit for gas exchange, the alveolus, are continuously subjected to cyclic mechanical stress arising from two interconnected systems: ventilation, with air coming in during inspiration and going out during expiration, and blood flow from the heart, which pumps de-oxygenated blood to the lung to recharge itself with O_2_. During lung development and growth – from late fetal life to somatic maturity – respiratory movements are essential for lung maturation together with forces coming from blood flow. The balance between the inward and outward forces of the rib cage impose mechanical stretch on lung parenchymal cells, allowing tissue growth and remodeling to relieve stress and strain (Bertoncello, 2015; Hsia, 2017). In adult life, the mechanical stresses coming from ventilation and blood circulation is essential for lung repair and regeneration, as these signals are transmitted from the surrounding environment to the lung cells and translated to molecular and biochemical processes that culminate in lung regeneration, repair, and remodeling (Bertoncello, 2015).

The pneumonectomy model has been proven useful for examining the interactions elicited by mechanical stimulation of the lung. Canine pneumonectomy experiments revealed a clear relationship between mechanical signals and lung remodeling. Following 42% resection, compensation for gas exchange mostly came from alveolar capillary recruitment and parenchymal remodeling (Carlin et al., 1991; Hsia and Johnson, 2006). Following 58% resection, alveolar and capillary growth occurred (Hsia et al., 1993; Bertoncello, 2015). Following 72% resection, the compensatory alveolar-capillary growth remained high, but functional compensation became limited by the heterogenous distribution of mechanical forces (Hsia et al., 2008). This pattern indicates that 58% resection could provide an optimal balance between lung remodeling and growth. Using high-resolution computer tomography at two inflation pressures and the deformation analysis, the same group showed that the remaining lobes had non-uniform regional mechanical strains. Two growth phases were identified: (i) an initial rapid proliferative phase, characterized by air space and blood volume increases, and (ii) a progressive remodeling phase, characterized by tissue relaxation and increased lung compliance persisting over several months (Hsia et al., 2008; Yilmaz et al., 2009).

While mammals cannot regenerate an entire lung, under appropriate stimuli they are able to add new gas exchange units, as exemplified by the compensatory lung growth during pneumonectomy (Ding et al., 2011; Bertoncello, 2015; Hsia, 2017). At microscale level, changes in the air and blood flow seem to provide a major stimulus for alveolar septation that starts at the thick side of the alveolar septum, giving rise to a new septum and therefore a new alveolus. The capillary growth of the new septum arises from existing vessels through intussusceptive angiogenesis, a process also supported by blood-borne bone marrow-derived CD34+ endothelial cells recruited into the lung vasculature, as observed during post-pneumonectomy lung growth (Hsia et al., 1993; Chamoto et al., 2012; Suga et al., 2013; Ackermann et al., 2014; Bertoncello, 2015; Hsia, 2017).

During regeneration of injured and diseased lungs, there are not always healthy mechanical stimuli to re-initiate lung growth. In all cases, a healthy vascular and perivascular microenvironment is necessary to establish “hospitable soil” for lung repair and regeneration, and to allow engraftment of therapeutic cells. Cao et al. (2017) showed that ectopic induction of endothelial cell-expressed angiocrine hepatocyte growth factor (HGF) and inhibition of perivascular NAPDH oxidase 4 (NOX4) in fibroblasts synergistically facilitated engraftment of murine and human ATII cells in different types of injuries mimicking lung fibrosis. Therapeutically, induction of the HGF-NOX4 axis could enhance the lung regeneration during injury and ameliorate the fibrosis or facilitate the engraftment of exogenous lung progenitors. Endothelial cells are also important at steady state, where they help self-renewal and differentiation of organ-specific stem cells (Rafii et al., 2016).

## Shear Stress in the Lung Vasculature

Blood vessels are tightly regulated by flow-mediated shear stress that is essential to maintain the quiescent state of the endothelial cells and to transmit mechanical signals that lead to phenotypic and functional changes in the vasculature, immune system, and lung parenchyma (Nonaka et al., 2014; Baeyens et al., 2016; Augustin and Koh, 2017; Hsia, 2017; Pellegata et al., 2018). Shear stress is of particular interest for regulating the phenotype and function of lung vasculature and the neighboring parenchymal cells. Endothelial cells are the first to sense the blood flow and the shear stress, and to transmit the signal to the surrounding cells. These mechanical signals involve the activation of receptors and subsequent biochemical signals.

Unlike other organs, the lung parenchyma does not rely on vascular perfusion to oxygenate its own cells, because of ventilation. In fact, O_2_ flows physiologically in the opposite direction, from the alveoli to the blood. Even static inflation of the lungs can maintain alveoli open and cells oxygenated for a reasonable time. Thus, reperfusion of the lung is different from that in other organs since O_2_, coming from the airway, is present during ischemia, and it is not reintroduced with reperfusion (Chatterjee et al., 2014).

To isolate the effects of only blood flow from those of O_2_, Fisher’s group has established an *in vivo* model of pulmonary ischemia (by stopping only blood flow) in isolated and perfused rat lungs, where constant alveolar PO_2_ (∼100 mmHg) was maintained by ventilation (Al-Mehdi et al., 1997; Song et al., 2001). Using this system, they identified that the cessation of blood flow was “sensed” by the endothelial cells and activated the “mechanosome” consisting of Platelet Endothelial Cell Adhesion Molecule (PECAM), VEGFR, and Vascular Endothelial (VE) cadherin (Chatterjee et al., 2014, 2015), to lead to the closure of K_ATP_ channels (Olesen et al., 1988), and activation of NADPH oxidase 2 (NOX2) to generate reactive oxygen species (ROS) and nitric oxide, ultimately leading to oxidative injury (Chatterjee et al., 2008; Browning et al., 2012). Endothelial depolarization also resulted in opening Ca^+2^ channels with increased intracellular Ca^ + 2^ and activation of nitric oxide (NO) synthase, increase in NO, and consequent vasodilation (Chatterjee et al., 2014). Other mechano-sensing molecules that have been identified include junctional proteins (VE-cadherin, occludin), receptor kinases (e.g., VEGFR2), integrins, focal adhesions (FAs), G-proteins, G-protein-Coupled Receptors (GPCRs), ion carriers and glycocalyx (Gulino-Debrac, 2013; Zhou et al., 2014; Chistiakov et al., 2017). Endothelial ROS-dependent signals facilitated binding and adhesion of neutrophils to the vascular wall, and those release ROS themselves causing inflammation and tissue damage (Gandhirajan et al., 2013; Menden et al., 2013).

In the ischemic environment, a combination of oxidative stress, exacerbated inflammation, and structural changes involving the endothelial cells (primary target) and lung parenchyma (secondary target) contributed to the declining lung function. Once lungs were again re-perfused, the return of blood flow led to further oxidant production, and the damage due to reperfusion exceeded that resulting from ischemia (Fisher et al., 1991). The onset of flow altered membrane polarity, similar to loss of shear with ischemia, suddenly increased shear with reperfusion triggered signaling that leads to NOX2 activation and ROS production. NOX2 activation in this case was driven via hyperpolarization that occured with onset of flow signaling (Lee et al., 2013a; Chatterjee et al., 2014).

Several events characterize I/R injury: epithelial injury/dysfunction, release of cytokines and damage-associated molecular patterns (DAMPs), and vigorous innate immune responses including activation of alveolar macrophages, invariant natural killer T cells and neutrophils. Most of these responses are initiated by rapid and robust generation of ROS that leads to cell/tissue injury, activation of multiple cell types, lipid membrane peroxidation and secretion of inflammatory cytokines and DAMPs (De Perrot et al., 2002; Andrade et al., 2006; Kreisel et al., 2010; Ferrari and Andrade, 2015; Laubach and Sharma, 2016; Zheng et al., 2017; Tatham et al., 2018). The activation and infiltration of innate immune cells, especially neutrophils into the graft during reperfusion is a key aspect of I/R injury largely driven by chemokines and ROS produced by donor lung cells such as epithelium, endothelium or macrophages that were injured and/or activated (Kobayashi, 2008; Gielis et al., 2015; Merry et al., 2015). Complications associated with increased ischemic time and reperfusion injury of transplanted lung grafts are responsible for decreasing the number of donor lungs suitable for transplantation and increasing the rate of primary lung graft failure and dysfunction (Christie et al., 2005; Diamond et al., 2013; Wang et al., 2019).

On the basis of these findings, it is clear that the altered cell signaling from abnormal blood flow (cessation of blood flow or reperfusion) affects the viability and overall quality of the lung. These factors are imperative to account for in the preparation of biological lung scaffolds for lung bioengineering or transplantation. The magnitude and distribution of mechano-transduction signals are critical for facilitating cell repopulation and engraftment, matrix remodeling, and re-establishment of the integrity of ACMs prior to *in vivo* implantation.

An approach to prevent lung I/R injury may be to maintain lung grafts within physiological conditions during procurement, preparation and storage to minimize the mechanotransduction signaling cascade initiated by ROS. In the rat lungs isolated *ex vivo*, it was observed that a drop in shear of >90% of the physiological flow was triggering the ischemia mechanosome (Al-Mehdi et al., 1998). Thus agents that could prevent the generation of ROS and NO, together with perfusion and ventilation of the lung grafts, for example *ex vivo* (see next section), may be a maneuver that prevent ischemia-reperfusion signaling and injury, significantly improving the quality of the lung grafts, the number of suitable organs for transplantation and reducing their failure post-transplantation.

## *Ex Vivo* Support and Recovery of Lungs for Transplant

**FIGURE 2:**
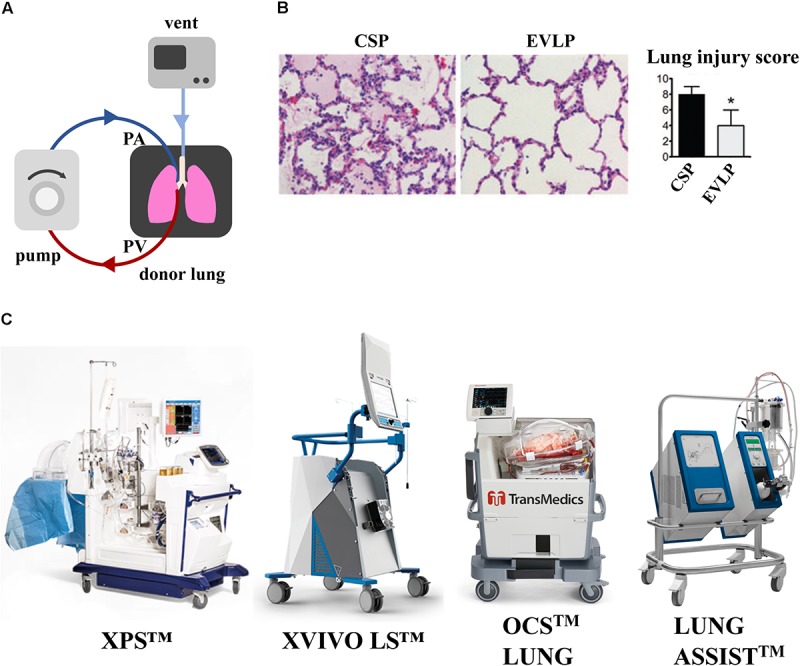
Effect of EVLP support on porcine lung ischemia/reperfusion injury. **(A)** Schematic representation of EVLP support. **(B)** Histologic analysis of lung biopsies of porcine lungs supported on EVLP showed less edema, hemorrhage and hyaline membranes, and lower lung injury score compared to static preservation perfusion (CSP) only. Images reproduced with permission from Cypel et al. (2009). **(C)** Commercially available EVLP devices for human lung support [XVIVO Perfusion System (XPS^TM^); XVIVO Lung System (LS^TM^),Organ Care System (OCS^TM^) and Lung Assist^TM^]. XPS^TM^ and XVIVO LS^TM^ are trademarks of XVIVO Perfusion AB; OCS^TM^ of TransMedics, Inc., Lung Assist^TM^ of Organ Assist Products B.V. All rights reserved. Pictures are published with permission from the above companies.

**FIGURE 3:**
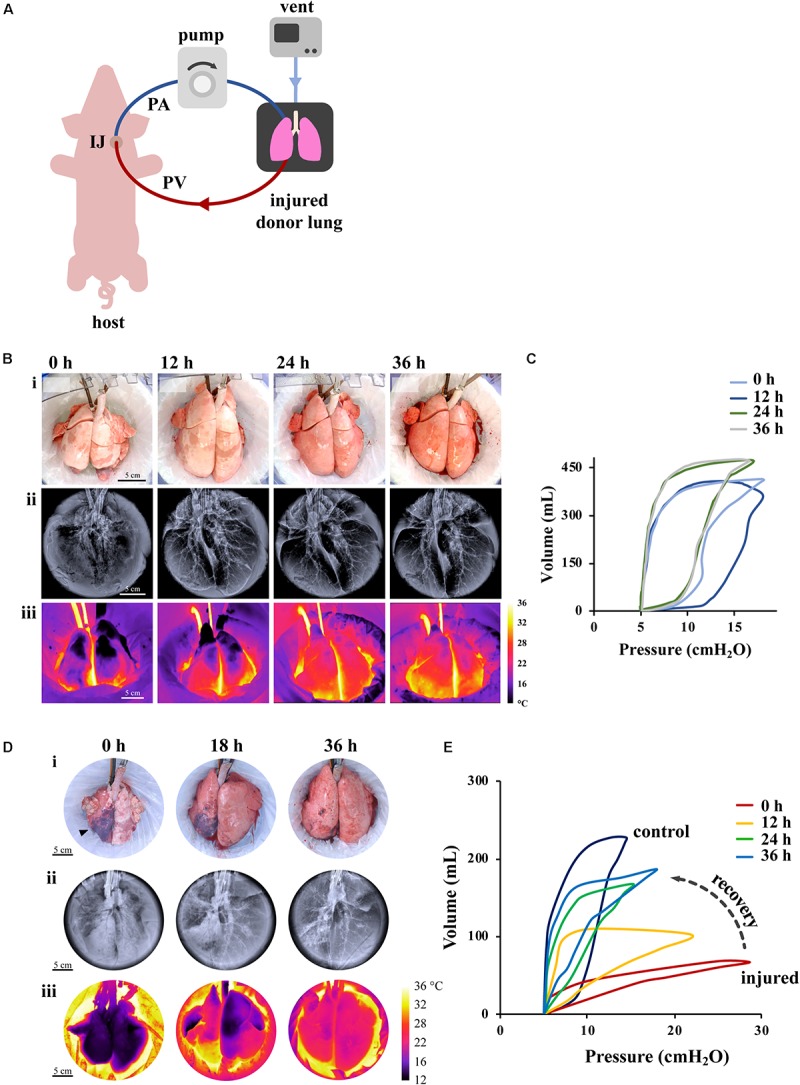
Effect of XC platform on porcine lung injury. **(A)** Schematic representation of XC platform. **(B,C)** Ischemic/reperfusion injury. **(B)** Porcine lungs mounted on XC platform showed macroscopic (i), radiographic (ii), and thermographic (iii) improvement after 18 h of cold ischemia. **(C)** Improvement of lung compliance of ischemic lungs on XC analyzed by pressure-volume loops (PV). Images reproduced with permission from O’Neill et al. (2017). **(D,E)** Gastric aspiration lung injury. **(D)** Porcine lungs mounted on 36 h XC platform showed macroscopic (i), radiographic (ii), and thermographic (iii) recovery after 6 h of porcine gastric contents injury. **(E)** Significant improvements of PV loops and dynamic compliance of injured lungs were observed throughout 36 h of XC support. Images reproduced with permission from Guenthart et al. (2019b).

A majority of donor lungs are discarded because of poor gas exchange function following episodes of hypoxia, injury, or infection. The two approaches currently being investigated for recovering those lungs, especially those of marginal quality, are *ex vivo* lung perfusion (EVLP) (Figure 2) and cross-circulation (XC) with a living host (Figure 3). In both cases, the goal is to provide lungs with physiologic conditions outside the body (through air ventilation, blood perfusion and metabolic clearance), in order to recover initially unusable donor lungs to a level suitable for transplantation. Both approaches are demonstrating potential to promote lung repair by ventilation and vascular perfusion, sometimes also augmented by mobilization of endogenous lung progenitors.

*Ex vivo* lung perfusion is the most common current approach. Hypothermic preservation that maintains organ viability by reducing cellular metabolism, does not facilitate lung tissue repair following the ischemic injury post-explant. Instead, EVLP support in normothermic conditions could provide prolonged preservation, but mostly, ameliorate the effects of ischemic injury and induce lung repair. Since its introduction by Steen et al. (2001), the EVLP platform has demonstrated noteworthy success at short-term support and partial recovery of marginal-quality donor lungs in both pre-clinical and clinical settings.

As shown in a porcine model of lung transplant, porcine lungs subject to 12 h cold static preservation (CSP) followed by 12 h of EVLP support versus 24 h of CPS, showed less interstitial edema, hemorrhage, cell infiltrate and lower lung injury score (LIS) (Figure 2B) (Cypel et al., 2009). A seminal study of prolonged EVLP (up to 12 h) was reported in 2008 using porcine and human lungs (Cypel et al., 2008), when the lungs were maintained in a physiological environment and recovered by circulating a perfusate enriched in O_2_ and electrolytes. This technology has been improved over time and was applied to human lungs by the Toronto Group in 2011, where 20 of 23 lungs initially deemed not suitable for transplantation were reconditioned on EVLP and transplanted into patients (Cypel et al., 2011). Although outcomes appear stable, concerns remain that suboptimal lungs will show higher rates of primary graft dysfunction and possibly inferior long-term survival (Botha et al., 2006). The most common EVLP devices used for human lungs are represented in Figure 2C. Current clinical protocols for EVLP limit perfusion time to 6 h, a duration of time practical for short-term support and recovery of certain marginal-quality lungs (e.g., edematous, ischemic). The Toronto Group has recently shown that marginal lungs reconditioned on EVLP have led to an increase in the number of patients undergoing transplantation, with comparable long-term outcomes to conventional lung transplantation (Divithotawela et al., 2019).

Over the years, EVLP used in research settings has enabled therapeutic cell delivery, airway lavage and surfactant replacement (Nakajima et al., 2017), and even the clearance and inactivation of hepatitis C virus (Galasso et al., 2019). While EVLP enables support and recovery of marginal-quality lungs up to 6 h (Cypel et al., 2011), the duration of lung support *ex vivo* is limited by the inability to provide the metabolic clearance and homeostatic physiologic environment of the lung, with its innate reparative mechanisms.

Long-term (i.e., multi-day) support is required for advanced bioengineering interventions (e.g., cell replacement therapy, gene therapy) that can recover *severely* injured lungs (most importantly those injured by gastric aspiration). Development of pulmonary edema, diminished gas exchange capability, and deterioration of the endothelial-epithelial barrier remain ubiquitous and formidable challenges that hinder the effectiveness of EVLP in providing long-term support. To address these limitations, our group recently introduced a novel XC platform for extracorporeal lung support using a large animal model (swine) (O’Neill et al., 2017; Guenthart et al., 2019b; Pinezich and Vunjak-Novakovic, 2019). In XC setting, the lung being recovered is placed into an EVLP-like organ chamber, ventilated in a normothermic, humidified environment, and connected to the blood perfusion with a swine recipient (Figure 3A) (O’Neill et al., 2017). Importantly, the swine recipient provides systemic regulation and metabolic clearance (renal, pancreatic, hepatic, etc.) that leads to full physiologic homeostasis that cannot be provided in the current EVLP settings.

Initial studies demonstrated the use of cross-circulation with an anesthetized swine host for support and recovery of injured donor lungs with a perfusion time of 36 h and a total preservation time greater than 60 h. In one study (O’Neill et al., 2017), two experimental groups were investigated: in one group (prolonged maintenance), healthy lungs were maintained on XC after circuit priming with donor blood; in a second group (ischemic recovery), lungs were first maintained at 4°C for 18 h to induce ischemic injury and then recovered on XC. We found that the lungs subjected to ischemia reperfusion showed reductions in lung edema after 4–6 h of support, by macroscopic, radiographic and thermographic parameters (Figure 3B). We also found that the pulmonary compliance (the ability of the lungs to stretch and expand) (Figure 3C), PaO_2_/FiO_2_ ratio, and tight-junction protein ZO-1 expression (a measure of alveolar–capillary permeability) decreased over the first 12 h, but then returned to the baseline by 24–36 h on cross-circulation. Notably, ischemic lungs recovered both functionally and morphologically, had decreased interstitial edema, restored the barrier function, and attained PaO_2_/FiO_2_ ratios measured in healthy lungs.

Based on the recovery of ischemic lungs on 36 h of XC, we investigated whether normothermic perfusion/ventilation of whole lungs *ex vivo* can be implemented for the functional recovery and regeneration of severely damaged lungs (Figures 3D,E). To this end, we established the clinically relevant swine model of lung injury by gastric aspiration, which is the most frequent cause of injury that renders donor lungs unsuitable for transplantation (Guenthart et al., 2019b). We investigated the ability of the XC platform to provide: (i) prolonged normothermic support of these lungs *ex vivo*, and (ii) lung regeneration over 36 h of XC with monitoring of the lung at the molecular, cellular, and tissue levels. The prolonged lung support enabled us to determine the effects of multiple therapeutic interventions: bronchoalveolar lavage, surfactant delivery, and alveolar recruitment.

Over 36 h of XC, injured lungs showed recovery of morphologic and functional aspects (Figures 3D,E), improvement in lung inflammation, measured by LIS, recovery of mechanical compliance to 68% of the average compliance of control lungs, a more than six-fold improvement. Significant changes in pressure–volume (PV) loops were consistent with this degree of recovery and did not resemble stereotypical PV loop hysteresis until after 24 h of cross-circulation (Figure 3E). Notably, following 36 h of XC there was no significant difference between the PaO_2_/FiO_2_ of injured and control lungs, demonstrating that even severely injured lungs can be recovered using this methodology (Guenthart et al., 2019b). Additionally, previous studies utilizing EVLP (Inci et al., 2008; Meers et al., 2010; Khalifé-Hocquemiller et al., 2014; Nakajima et al., 2017) have investigated recovery of global lung function (e.g., compliance, gas exchange), but not the dynamic interplay between cellular metabolism, activity and regeneration of lungs while on extracorporeal support as with our XC platform.

Recognizing that more advanced bioengineering therapies would necessitate even longer perfusion times, we next sought to further extend the XC timeline. In our most recent studies, we demonstrated that the duration of XC for normothermic extracorporeal lung support in a swine model can be extended to 100 h (Hozain et al., 2019). This model provided the maintenance of the lung *ex vivo* that was connected to an awake swine host, with full hemodynamic stability. Throughout 4 days of normothermic support, the function of extracorporeal lungs was robustly maintained (PaO_2_/FiO_2_ > 400 mmHg; compliance > 20 mL cmH_2_O^–1^), and recipient swine were hemodynamically stable (lactate <3 mmol L^–1^; pH 7.42 ± 0.05). Radiography confirmed extracorporeal lungs remained aerated, and bronchoscopy revealed normal airways without edema or secretions. Analysis of bronchoalveolar lavage fluid revealed no significant increases in inflammatory cytokines from baseline to day 4. Histologic evaluation showed an intact blood-gas barrier and full preservation of the airway and alveolar architecture as well as cellular viability and metabolism over 4 days of normothermic support. While future studies will need to investigate and confirm that this approach is robust and reproducible across large numbers of lungs with different types and stages of injury, and a wider variety of experimental conditions, we anticipate that this model can be extended into a human model of awake extracorporeal lung recovery. The use of a multi-day XC platform could enable recovery of damaged lungs not currently salvageable using EVLP systems with the ultimate goal to recover the functionality of the lung and reduce the risks of primary graft dysfunction often due to lung injuries that occurred from the time of brain death to reperfusion in the recipient (Christie et al., 2005; Chaney et al., 2014). Furthermore, what really makes an impactful difference of XC from other *ex vivo* supports such as EVLP, is the length of physiologic lung support that enables proper assessment of the lung graft and allows investigation of bioengineering strategies to improve lungs before transplantation with therapeutic interventions such as gene or cell therapies.

## Lung Bioengineering by Decellularization and Recellularization

Tissue engineering aims at replacing or regenerating human tissue with the final goal of restoring normal function, through an integrated use of cells, signaling molecules, and scaffolds (Gilpin and Yang, 2017). To provide the main function of lung, gas exchange, lung bioengineering has attempted for years the use of synthetic hollow-fiber membranes, oxygenators, as lung substitutes. An ideal implantable lung should be flexible in size, performing acceptable gas exchange and maintaining good biological compatibility. Unfortunately the existing synthetic membranes have several limitations including: (i) *Size*, as the membrane devices supplying the same exchange area as a human adult (70–100m^2^) could not be contained within an average adult body size. (ii) *Limited-time performance* as they last only for days to weeks; (iii) *Thrombogenicity*, as these membranes are made of synthetic material and easily activate the recipient immune system and the intravascular coagulation, requiring life-long anticoagulation. Several groups are investigating the optimal strategies to cover those membranes with autologous endothelial cells to improve their haemocompatibility and build a “bio-hydrid” system, but the clinical reality of a long term lung substitute remains far at the moment (Polk et al., 2010; Wagner and Griffith, 2010; Zwirner et al., 2018; Young and Dilling, 2019).

Over the last two decades, these issues with synthetic lung substitutes have led to an increasing interest in utilizing biological scaffolds, which maintain the structural, biomechanical, and biochemical properties of the native organ and therefore can guide cells to reconstitute the physiological function of the organ. These features of biological scaffolds have turned interest to lung decellularization, where all cellular material is removed from the lung and only the native scaffold, made of ECM, is left behind. Four criteria need to be satisfied to obtain optimal ECM after decellularization: (i) <50 ng double-stranded DNA (dsDNA) per mg of decellularized material; (ii) less than 200 bp length of DNA fragments; (iii) preservation of structural proteins of ECM; and (iv) retention of mechanical properties (Gilpin and Yang, 2017). Particularly important in reducing the scaffold immunogenicity is the complete removal of genetic material (Crapo et al., 2011). Of course, the optimal removal of DNA does not come without pitfalls. In fact, harsh decellularization can damage ECM’s microstructure and ultrastructure, making recellularization difficult and incomplete.

Two groups pioneered lung decellularization to obtain a scaffold for seeding primary epithelial into the airways and endothelial cells into the vascular compartment, to enable restoration of gas exchange (Ott et al., 2010; Petersen et al., 2010). However, these bioengineered lungs failed after only a few hours upon transplant, due to the incomplete regeneration of vasculature that remained leaky and resulted in alveolar edema and thrombosis (Ott et al., 2010; Petersen et al., 2010, 2011, 2012; Song et al., 2011). Typically, fully decellularized lungs are used as scaffolds for seeding of epithelial and endothelial cells (Ott et al., 2010; Petersen et al., 2010; Wallis et al., 2012; O’Neill et al., 2013; Wagner et al., 2014). Because human lungs contain more than a billion cells, complete decellularization and recellularization may be impractical at the clinical scale. Unlike some other tissues (e.g., blood vessels and bones), lungs cannot be grown using cells on synthetic scaffolds, due to the structural and biological complexity of the parenchyma and vasculature and the need for many different cell types to reconstruct such a complex organ. Lung regeneration using a completely decellularized lung repopulated with epithelial and vascular cells remains slow and incomplete, due in large part to the fact that the lung contains more than 40 different cell types (Colby et al., 2007; Franks et al., 2008; Beers and Morrisey, 2011; Wagner et al., 2013). Furthermore, endothelial cells have different phenotypes in the arterial and venous compartments, making the recellularization of lung vasculature extremely complex. A problem encountered with this approach was in the incomplete endothelial coverage of the scaffold that resulted in blood clotting, hemorrhage, and edema in the lung airway. Ott’s group improved their protocol by co-seeding endothelial cells with mesenchymal cells and by delivering cells into the vascular tree from both the arterial and venous sides of the lung. Under these conditions, the endothelium coverage increased to 75%. While the vasculature in these regenerated lungs was still leaky, they maintained patency *in vivo* for the whole 3 days (Ren et al., 2015).

## Lung Bioengineering by Selective Replacement of Epithelium

Lung epithelium is not only the main target of lung diseases, congenital and acquired, but also the most critical component involved in lung repair and functional recovery. There are several examples demonstrating the critical role of epithelium in the initiation of parenchymal lung disease, including monogenic disease such as Hemansky-Pudlack syndrome, Niemann-Pick disease, surfactant proteins defects (*SFPTA1*, *SFPTA2*, *SFPTB*, *SFTPC*), and mutations in the ABC subfamily 3 (*ABCA3*) (Beers and Morrisey, 2011). These diseases are characterized by dysfunctional ATII cells displaying fibrotic phenotype. In mice, targeted ablation of ATII cells results in extensive lung fibrosis (Sisson et al., 2010; Kim et al., 2018). Expression of mutant forms of *SFTPC* associated with human respiratory disease show ATII cell injury, such as endoplasmic reticulum (ER) stress and apoptosis (Mulugeta et al., 2007; Korfei et al., 2008; Maitra et al., 2010; Lawson et al., 2011; Katzen et al., 2019). *SFTPC* and *ABCA3* mutations are associated with idiopathic interstitial pneumonia shown to induce apoptosis of epithelial cells *in vitro* and *in vivo* (Glasser et al., 2003; Bullard et al., 2005; Wert et al., 2009; Rindler et al., 2017). Epithelial injury is also a central finding in the lungs of patients with the acute respiratory distress syndrome (ARDS) patients.

Extensive epithelial damage is often involved in the loss of the epithelial-mesenchymal homeostasis, with rearrangement of ECM and lung architecture leading into fibrosis. Reconstitution of functional epithelium (re-epithelialization) is crucial for preventing pathological lung remodeling and for recovering the most important lung function: gas exchange. Hypothetically, regeneration with healthy epithelial cells could promote local proliferation of the remaining undamaged epithelium, activated by the local lung progenitor or use of exogenous stem cells (Beers and Morrisey, 2011).

**FIGURE 4:**
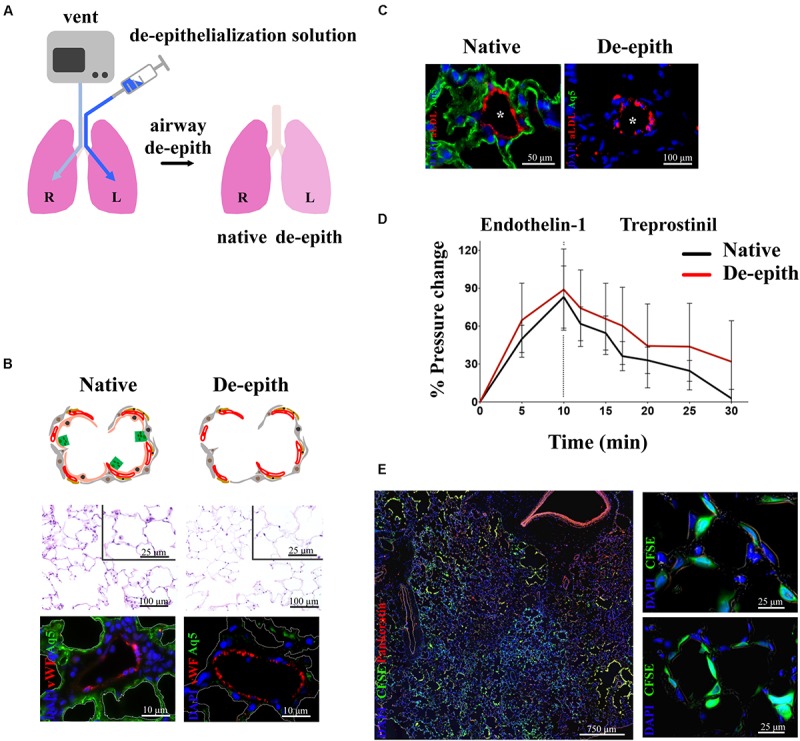
Removal of lung epithelium with preservation of functional vasculature. **(A)** Schematic representation of single lung de-epithelialization on EVLP (R lung control, L lung treated). **(B)** Histological analysis showing removal of lung epithelium in the respiratory zone (upper panel). Immunostaining showing removal of alveolar Type I cells (Aq5) and preservation of endothelial cells (vWF) (lower panel). **(C)** Endothelial viability showed by uptake of Ac-LDL by endothelial cells in de-epithelialized lung. **(D)** Preservation of vasoresponsiveness of de-epithelialized lung to Endothelin-1 and Treprostinil. **(E)** De-epithelialized rat lungs recellularized with CFSE-labeled human SAECs. Attachment of CFSE-labeled SAECs in the alveoli (higher magnification panels). Images reproduced with permission from Dorrello et al. (2017).

Given that the vascular component of the lung is critical for the supply of nutrients and O_2_, much effort has been invested into finding the best strategies to repair lung epithelium while keeping the vascular network intact and functional. The derivation and use of vascularized lung scaffolds has been pioneered by our group (Dorrello et al., 2017) (Figure 4). Since lung epithelium is often damaged by lung disease, our group developed an airway-specific approach to remove the pulmonary epithelium, de-epithelialization, while maintaining the viability and function of the vascular endothelium, using a rat model mounted on EVLP platform (Figure 4A). The alveolar region of the lung was efficiently de-epithelialized as shown by reduction of ATI marker, Aquaporin 5, with preservation of endothelial cells (vWF) (Figure 4B). Viability and function of endothelial cells were shown by the capture of acetylated LDL (Figure 4C). Blood vessels maintained responsiveness to vasoconstrictor/dilators after de-epithelialization (Figure 4D). The resulting vascularized lung grafts supported the attachment and growth of human adult pulmonary cells and stem cell–derived alveolar progenitor cells after 48 h of bioreactor culture (Figure 4E) (Dorrello et al., 2017).

Our de-epithelialization approach has been designed to overcome three major limitations of the whole lung decellularization: (i) the challenging hurdle to properly recellularize an organ consisting of 40 distinct cell types (Colby et al., 2007; Franks et al., 2008; Beers and Morrisey, 2011; Wagner et al., 2013); (ii) the lack of functional vascular network with the relative high risk of thrombogenicity, pulmonary edema and hemorrhage within the lung graft (Ott et al., 2010; Petersen et al., 2010, 2011, 2012; Song et al., 2011; Ren et al., 2015); (iii) the paucity of all supporting cells (interstitial and vascular) that through delivery of growth factors and signaling molecules could foster appropriate lung epithelial regeneration.

To enable replacement of the epithelium in specific local areas of the lung (rather than in the entire lung) while maintaining intact vascular perfusion and endothelial lining (as opposed to removing both, epithelial and endothelial cells), our group developed a methodology for targeted delivery of microliter volumes of decellularization fluid or cell suspension into the lung from the upper airway all the way to the most distal alveolar spaces (Kim et al., 2015, 2017). In this approach, a soluble liquid plug of very small volume (<1 mL) is introduced into the upper airway. Using programmed air ventilation of the lung, the plug is pushed into a specific area of distal airway to achieve deposition of liquid film onto the lung epithelium. To demonstrate the utility of this method in a clinically relevant model, we utilized human lungs rejected for transplant and healthy porcine lungs *ex vivo*. In both cases, lungs were maintained on air ventilation and vascular perfusion. We demonstrated that this method could be used for decellularization and lavage needed to remove epithelial cells, and also for delivering therapeutic cells to the denuded locations in the airway.

**FIGURE 5:**
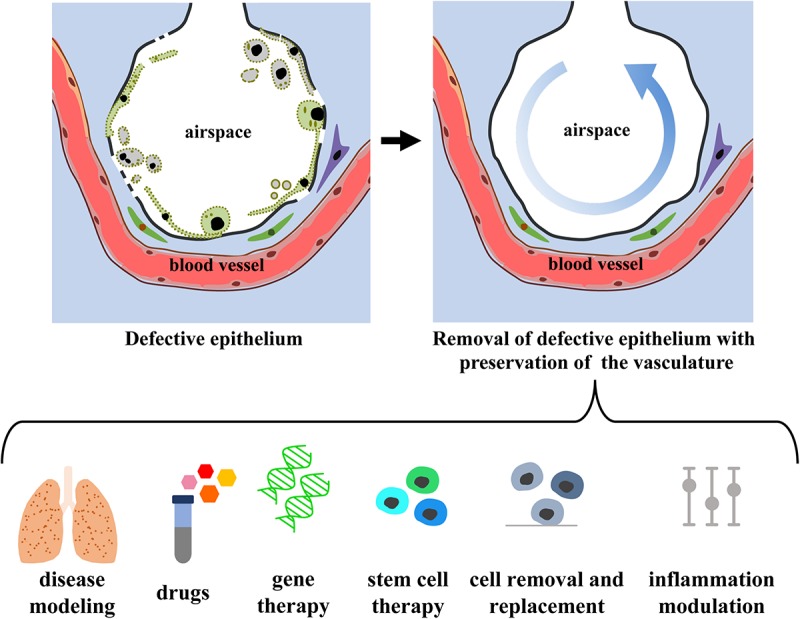
Envision of diagnostic and therapeutic interventions of a functional vascularized lung graft. Schematic representation of lung de-epithelialization **(upper panel)** and potential diagnostic and therapeutic interventions **(lower panel)**.

De-epithelialized lungs with intact, functional vasculature could serve as a physiologic scaffold by: (i) enabling the delivery of O_2_, nutrients, growth factors, and signaling molecules, (ii) providing biophysical and mechanical signals via perfusion (flow, shear) and ventilation (strain), and (iii) maintaining the ECM (biochemical moieties, adhesion molecules, matricryptic peptides) and the interstitial and support cells (fibroblasts, pericytes, endothelial, mesothelial, and lymphatic cells). De-epithelialized lung scaffolds could be repopulated with embryonic, fetal, adult primary or induced pluripotent stem cells, while maintaining functional vascular and interstitial compartments, and provide a valuable physiological model for investigating: (i) lung development, (ii) etiology and pathogenesis of lung diseases involving the pulmonary epithelium, (iii) acute lung injury and repair, and (iv) drug and cell therapies (Figure 5). Lung scaffolds with intact vascular network may also allow for recellularization using patient-specific cells, obtaining chimeric lungs capable of gas exchange and potentially able to match the extended criteria for lung transplantation.

## Therapies Based on the Delivery of Cells and Cell Products

The shortage of transplantable donor lungs for patients with end-stage lung disease has motivated scientists to investigate alternative therapeutic strategies to traditional transplantation. Cell-based therapy has dominated the lung field in the last two decades. The main strategy has been to generate a functional lung graft by combining a natural lung scaffold with appropriate cell types to create a transplantable bioengineered lung.

In 2010, the studies conducted by Niklason’s and Ott’s groups on whole lung decellularization and recellularization *ex vivo*, demonstrated the capability of a decellularized lung graft to support engraftment of multiple cell types, despite the fact that alveolar edema and thrombosis caused lungs to fail after only a few hours following transplant (Ott et al., 2010; Petersen et al., 2010, 2011, 2012; Song et al., 2011). These studies and other meritorious work (Gilpin et al., 2014; Nichols et al., 2014, 2018; Wagner et al., 2014; Zhou H. et al., 2018) emphasized three major developments in the field of lung bioengineering: (1) the capacity of a properly conditioned lung scaffold to facilitate cell engraftment; (2) the utilization of *ex vivo* devices, such as EVLP, to support, assess, and optimize lung grafts; (3) the possibility to intervene with cell therapy in lung grafts supported *ex vivo*. However, these advances also posed a major challenge to researchers: to create a graft with the functional capability of the lung, an extremely complex organ containing more than 40 different cell types (Colby et al., 2007; Franks et al., 2008; Beers and Morrisey, 2011; Wagner et al., 2013).

Methods for optimization of lung scaffolds have radically improved over time to promote cell engraftment (Dorrello et al., 2017; Gilpin and Yang, 2017). Additionally, advances in the field of *ex vivo* whole organ bioreactors allowed for extended support of lung grafts outside the body using either EVLP (Steen et al., 2001; Wierup et al., 2006; Cypel et al., 2008, 2011; Divithotawela et al., 2019) or the XC platform pioneered by our group (O’Neill et al., 2017; Guenthart et al., 2019b; Hozain et al., 2019). Recognizing the challenge of repopulating a fully denuded scaffold with the many cell types critical to lung function, researchers have shifted from whole recellularization to a more targeted approach. Specifically, targeted removal of certain cell populations most relevant in lung diseases provides a means of treating the condition without the need to exchange *all* the cells in the lung. Since the discovery of the induced pluripotent stem cells (iPSCs) in 2006 (Takahashi and Yamanaka, 2006), there has been an increasing interest in the use of autologous cell therapy for several organs, including lung. In fact, these cells could be directly generated from the recipient and expanded *in vitro* to provide an unlimited supply of cells without the need for embryonic tissues. Furthermore, the possibility to genetically reprogram somatic cells could allow for correction of mutations in patient-specific progenitor cells. In case of lung disease, these cells can be used in a cell replacement therapy, where endogenous (injured or diseased) cells are removed and subsequently replaced with healthy, engineered lung progenitors. Cell type, number, engraftment and delivery strategies (airway versus intravenous delivery) are important factors that have been explored in recent years.

A targeted cell-based therapy that replaces only the defective or injured cells while preserving the surrounding lung matrix and supporting cells would be ideal. Since most lung diseases primarily affect the epithelium (Table 1), major efforts have been made to expand human iPSC-derived lung epithelial cells *in vitro* for differentiation to mature cell types (proximal versus distal fate) prior to delivery in the lung (Huang et al., 2014; Chen et al., 2017; McCauley et al., 2017; Berical et al., 2019). The first report of human iPSC-derived lung epithelial cell engraftment in the lung *in vivo* was described in murine model of lung injury in 2018 (Miller et al., 2018). This study demonstrated the feasibility of using animal models to investigate the optimal conditions for cell delivery *in vivo*, but it also emphasized the need for physical space within the epithelial niche to allow for attachment of newly introduced cells. In this case, a chemical agent (naphtalene) was used to induce epithelial injury in the airways and remove endogenous cells to make room for new cells to engraft. Several other groups have shown engraftment of murine or human lung stem cells in different lung injury models in mouse, including naphthalene, radiation, detergent, and virus mediated injury (Leblond et al., 2009; Rosen et al., 2015; Vaughan et al., 2015). The common theme to all these models is that the lung injury serves as a means of creating space for therapeutic cells to engraft.

In fact, the ideal strategy to deliver therapeutic cells and promote their engraftment is to gently remove and replace defective cells with healthy ones, while preserving native lung vasculature and ECM, components that can support the newly introduced cells by providing a favorable lung cell niche. Specifically, lung vasculature ensures that the delivered cells receive all nutrients and factors to properly engraft. Additionally, shear forces from blood flow and strain forces in the ECM during normal breathing provide mechanical stimuli to the cells. In line with these targeted therapeutic strategies, we have applied regional de-epithelialization *ex vivo* in small animal model on EVLP (Dorrello et al., 2017) and in large animal model on EVLP and on XC platform (Kim et al., 2017; O’Neill et al., 2017). This allowed for attachment and engraftment of human adult pulmonary cells and stem cell-derived alveolar progenitor cells. As proof of concept, in rodent lungs de-epithelialized and supported on EVLP, we introduced via airway human iPSC-derived lung epithelial cells and showed attachment to the native ECM of distal lung and expression of ATI and ATII cell markers (Dorrello et al., 2017). In conditions such as ARDS, with an overwhelming inflammation, mesenchymal stem cells (MSCs) could be added to ameliorate lung injury and facilitate re-epithelialization. In this contest, we have showed integration of labeled MSCs delivered into the distal lung regions via intratracheal liquid instillation in rat, human and porcine lungs on EVLP and XC platforms (Kim et al., 2017; O’Neill et al., 2017; Guenthart et al., 2019a).

In diseases affecting predominantly conductive airways, such as cystic fibrosis, targeted de-epithelialization of the conductive airways could allow removal of the mutant transmembrane conductance regulator (*CFTR*) epithelial cells and replacement with normal human bronchial epithelial cells or iPSCs where *CFTR* mutation is corrected (Cutting, 2015; Berical et al., 2019). Furthermore, extended lung support on XC up to four days that has been recently shown by our group (Hozain et al., 2019), could allow sufficient time for engraftment and differentiation of delivered cells prior to transplantation into a recipient patient.

In addition to human iPSC-derived lung epithelial cells, another cell type that has been well studied is the MSC. MSCs are non-hematopoietic stromal cells that express low levels of major histocompatibility complex (MHC) type I and lack MHC type II and T-cell costimulatory molecules, making them highly non-immunogenic. Additionally, MSCs can be rapidly expanded *in vitro* and have important immune-inflammatory modulatory effects on the surrounding cells to make them ideal candidates to promote organ repair, including in the lung (Geiger et al., 2017; Huppert et al., 2019; Ross et al., 2019). These cells have been investigated in lung injury models *in vivo* and in lungs supported by EVLP or XC (Lee et al., 2009, 2013b; McAuley et al., 2014; Kim et al., 2017; O’Neill et al., 2017). Lee et al. (2009) showed that administration of MSCs reduced lung endothelial injury and restored alveolar fluid clearance to normal levels in human lungs injured with *E. coli* endotoxin supported on EVLP. *In vivo*, it has been demonstrated that intratracheal administration of human bone marrow–derived MSCs reduced bacterial growth in the lungs of mice with *E. coli* pneumonia (Krasnodembskaya et al., 2010). In ARDS, MSCs are currently being tested in phase I and II clinical trials with excellent safety profile (Wilson et al., 2015; Matthay et al., 2019), as well as in IPF (Tzouvelekis et al., 2013; Chanda et al., 2016; Fishman et al., 2019).

The mechanisms by which MSCs exert their therapeutic benefits appear to be through paracrine activity, mitochondrial transfer, and extracellular vesicles, rather than through engraftment in the airway (Huppert et al., 2019). MSC treatment of *E. coli* toxin injured human lungs on EVLP reduced lung weight gain, improved lung endothelial barrier permeability, and restored alveolar fluid clearance in a Fibroblast Growth Factor (FGF) dependent manner (Lee et al., 2009). In a mouse model of lung injury, Bhattacharya’s group showed that bone marrow–derived MSCs transfer red mitochondrial DNA to host alveolar cells restoring their bioenergetics and surfactant secretion, while reducing mortality (Islam et al., 2012). Administration of MSC microvesicles in lungs on EVLP led to improved alveolar fluid clearance and reduced bacterial load in human lungs with *E. coli* pneumonia (Park et al., 2019), and decreased weight gain and improved airway and hemodynamic parameters in human lungs rejected for transplantation (Gennai et al., 2015).

In diseases involving both cellular malfunction and highly inflamed microenvironments, multiple cell types may need to be administered. In this scenario, replacing the damaged epithelial cells with healthy ones along with co-delivery of MSCs could potentially augment cell engraftment, lung repair, and recovery. This co-administration of cell types could be combined with other strategies such as targeted de-epithelialization and/or prolonged support offered by EVLP and XC, to promote lung regeneration.

In terms of cell number, because human lung contains roughly 1 × 10^11^ pneumocytes and 70 × 10^9^ endothelial cells (Crapo et al., 1982; Mercer et al., 1994), the initial approach to replace all these cells in human is extremely challenging in terms of cell types and numbers. Bone marrow transplant, widely used to treat hematopoietic diseases, suggests that even low level of chimerism can effectively reverse the disease phenotype (Abraham et al., 2017; Fitzhugh et al., 2017). Similarly, in the lung, some studies suggest that not all defective or injured cells need to be replaced to improve the lung disease in question. For example, functional recovery of the CF lung may be achievable if CFTR levels are restored to even 15–20% of normal airway expression (McKone et al., 2003). As for cell-based therapy in other organs, several aspects still warrant further investigation, including the selection of an optimal cell type to be delivered, optimal source of cells, route of administration, number of doses, and safety of these therapies. *Ex vivo* organ support systems such as EVLP and XC could aid in addressing these questions.

## Conclusion

Lungs remain the most under-utilized solid organ for transplantation, with as many as 80% donor lungs rejected for transplant. In most cases, the injury is located in the alveolar epithelium, affecting the main and vital function of the lung, the gas exchange, and not meeting the standards for transplantation. Advances in EVLP have enabled the evaluation and reconditioning of marginally unacceptable donor lungs. Extending the duration of extracorporeal support from hours to days would enable the implementation of lung bioengineering approaches, such as cell replacement in the donor lung, that would expand the pool of donor lungs and improve the long-term outcomes of lung transplantation. An approach currently studied, using the clinical-scale swine model, involves XC with a recipient, resulted in up to 100 h of normothermic perfusion to maintain and recover lungs for transplant through multiscale therapeutic interventions. These lungs exceeded transplantation criteria, while not causing any significant changes in physiologic parameters.

Lung bioengineering requires a delicate balance between removing the cells and preserving the matrix make-up and functionality. Decellularization methodologies are always associated with some level of damage to the matrix. The attempts to fully recellularize decellularized lungs still do not result in functional lungs, due to poor vascularization, lack of proper organotypic endothelial cells, and inability to reconstruct the high complexity of the lung. Our group opted for a de-epithelialization approach, as a more gentle method to potentially remove most of the diseased epithelial cells, leaving intact the ACM and the lung’s complex vascular network. Decellularization of diseased lungs, such as those afflicted by COPD, emphysema, BPD, or IPF, would provide opportunities to study the timing of disease progression and altered signaling between the epithelial and vascular compartments – leading to the possible identification of drugs that could effectively restore physiological epithelial-mesenchymal-vascular homeostasis in diseased lungs.

In the stem cell field, a challenge is to identify the key ACM factors that could modulate alveolar stem cell function and regulate lung scaffold repopulation, including, for example, different subtypes of metalloproteases, serine proteases, and matrisome proteins that could act through integrins and augment lung progenitors’ regeneration. Vascularized lung scaffolds with intact ACM could be applied to identify the proper ACM signals and define the “ACM regenerative profile.” Cocktails of factors (e.g., angiocrine and regulatory factors) could also be used to specifically enrich decellularized lung scaffolds prior to seeding with lung progenitor cells, with the goal to achieve lung regeneration and repair under increasingly physiological conditions. An “ideal” scaffold for lung tissue engineering, obtained by de-epithelialization of the lung, would maintain: (i) functional ACM for lung progenitor cells to adhere, engraft, and differentiate, (*ii*) intact perfusable and viable vascular network, and (iii) regional specificity to allow seeded lung progenitors to migrate to their specific natural niches and differentiate into site-specific lineages.

## Data Availability Statement

The raw data supporting the conclusions of this article will be made available by the authors, without undue reservation, to any qualified researcher.

## Ethics Statement

The studies conducted by our group complied with the relevant ethical regulations for animal testing and research. Approval for this study was received from the Institutional Animal Care and Use Committee at Columbia University. Animal care and procedures were conducted in accordance with the US National Research Council of the National Academies *Guide for the Care and Use of Laboratory Animals*, 8th Edition.

## Author Contributions

ND and GV-N jointly planned and wrote the manuscript.

## Conflict of Interest

The authors declare that the research was conducted in the absence of any commercial or financial relationships that could be construed as a potential conflict of interest.
